# Repertoire of free-living protozoa in contact lens solutions

**DOI:** 10.1186/s12886-016-0370-6

**Published:** 2016-10-29

**Authors:** Ibtissem Bouchoucha, Aurore Aziz, Louis Hoffart, Michel Drancourt

**Affiliations:** 1 Pôle des Maladies Infectieuses, Hôpital de la Timone, Marseille, France; 2 Ophthalmology Department, Hôpital de la Timone, Marseille, France; 3 Aix Marseille Université, URMITE, UM63, CNRS 7278, IRD198, Inserm 1095, 13005 Marseille, France; 4 Unité de Recherche sur les Maladies Infectieuses et Tropicales Emergentes, Faculté de Médecine, 27, Boulevard Jean Moulin, Marseille cedex 5, France

**Keywords:** *Acanthamoeba*, Keratitis, Contact lens solution, Protozoa

## Abstract

**Background:**

The repertoire of free-living protozoa in contact lens solutions is poorly known despite the fact that such protozoa may act as direct pathogens and may harbor intra-cellular pathogens.

**Methods:**

Between 2009 and 2014, the contact lens solutions collected from patients presenting at our Ophthalmology Department for clinically suspected keratitis, were cultured on non-nutrient agar examined by microscope for the presence of free-living protozoa. All protozoa were identified by 18S rRNA gene sequencing.

**Results:**

A total of 20 of 233 (8.6 %) contact lens solution specimens collected from 16 patients were cultured*. Acanthamoeba* amoeba in 16 solutions (80 %) collected from 12 patients and *Colpoda steini*, *Cercozoa* sp., *Protostelium* sp. and a eukaryotic more closely related to *Vermamoeba* sp., were each isolated in one solution. *Cercozoa* sp., *Colpoda* sp*.*, *Protostelium* sp. and *Vermamoeba* sp. are reported for the first time as contaminating contact lens solutions.

**Conclusion:**

The repertoire of protozoa in contact lens solutions is larger than previously known.

## Background

Contact lens (CL) wearers are at risk of developing infectious keratitis [1]. In particular, the prevalence of amoebic keratitis has been shown to be significantly higher in CL wearers than in the general population living in the same geographic area [2]. Accordingly, it has been suspected that CL solution could be the source of amoeba in this situation [3]. Indeed, several studies have reported detecting amoeba in CL solutions [2]. Thus far, only amoeba of the genus *Acanthamoeba* have been documented in CL solutions [1, 4, 5].

Here, we prospectively search for free-living unicellular protozoa in CL solutions collected from patients with suspected keratitis, in an effort to broaden the repertoire of free-living protozoa as potential cornea pathogens.

## Methods

### Culture of protozoa

CL solution specimens were collected between 2009 and 2014 by CL wearers presenting to the Ophthalmology Department of the Timone Hospital in Marseille, France, for the clinical diagnosis of keratitis and corneal ulcers. Clinical criteria for diagnosis included evidence of a corneal infiltrate or corneal ulcer with underlying inflammation, which could lead to the necrosis of corneal tissue. CL solution provided by the patient was poured into a sterile can kept at room temperature for 4–24 h before it was analysed in the laboratory. The following standard protocol was used to search for protozoa. The CL solution was spread onto a non-nutrient agar plate, plated with a lawn of living *Enterobacter aerogenes*. The non-nutrient agar plate was incubated at 28 °C in a humidified atmosphere (contact with moistened gauze) and examined by microscope at × 4 and × 10 magnifications. Free-living protozoa were subcultured on a new non-nutrient agar plate with living *E. aerogenes* in order to obtain sufficient clonal populations. When the growth was sufficient, areas where protozoa were easily detected by microscope were cut and centrifuged at 2000 g for 10 min. The pellet was re-suspended in 1 mL of Amoeba Page’s saline PAS (Dunstaffnage Marine Laboratory, Oban, UK) for further DNA extraction.

### Culturing bacterial and fungal organisms

CL solution specimens were seeded onto 5 % sheep-blood agar (COS, bioMérieux, La-Balme-les-Grottes, France) and BCYE (Buffered Charcoal Yeast Extract, bioMérieux) and incubated at 32 °C for 10 days in a 5 % CO_2_ atmosphere. For the culture of yeasts and fungi, CL solution specimens were seeded onto Sabouraud agar containing chloramphenicol and gentamicin (bioMérieux), incubated at 32 °C for 10 days. All the bacterial isolates were identified using matrix-assisted laser desorption ionization-time of flight mass spectrometry (MALDI-TOF-MS; Microflex, Bruker Biospin S.A., Wissembourg, France) as previously described [6]. Briefly, colonies detached from the agar were directly applied to a MALDI-TOF MTP 384 target plate (Bruker) in order to analyze four spots per isolate. Each spot was overlaid with 2 μL of matrix solution, a saturated solution of α-cyano-4-hydroxycinnamic acid in 50 % acetonitrile mixed with 2.5 % tri-fluoracetic-acid. The matrix-sample was crystallized by air-drying at room temperature for 5 min. Measurements were performed using an Autoflex II mass spectrometer (Bruker Daltonik) equipped with a 337-nm nitrogen laser. Spectra were recorded in the 2–20 kDa mass range. Data were automatically acquired using AutoXecute acquisition control software. The two first raw spectra obtained for each isolate were imported into the BioTyper software, version 2.0 (Bruker Daltonik GmbH), and were analyzed by standard pattern matching (with default parameter settings) against 5625 references in the BioTyper database. When both spots yielded a score ⩾1.9, identification was complete. In this study, it was not necessary to complete accurate MALDI-TOF-MS identification of bacteria by DNA sequencing.

### Molecular identification of protozoa

Total DNA was extracted using the QIAmp tissue kit according to the manufacturer’s protocol (QIAGEN SA, Courtaboeuf, France). A 328-bp fragment of the 18S rRNA gene was PCR-amplified using the primers NS5/F 5′AACTTAAAGGAATTGACGGAAG3′ and NS6/R 3′GCATCACAGACCTGTTGCCTC5′ and an annealing temperature of 60 °C [7]. All amplification reactions were performed using the 2720 thermal cycler (Applied Biosystems, Saint-Aubin, France) in a 50 μL-mixture containing 5 μL of dNTPs (2 mM of each nucleotide), 5 μL of DNA polymerase buffer (Qiagen), 2 μL of MgCL2 (25 mM), 0.25 μL HotStarTaq DNA polymerase (1.25 U) (Qiagen), 1 μL of each primer and 35.75 μL of DNAse-free water. The positive control consisted of *Candida albicans* DNA. Sterile distilled water was used as a negative control. PCR consisted of a 15-min initial denaturation *Taq* polymerase Hot-Star at 95 °C followed by 30-s denaturation at 95 °C, 30-s hybridation at 60 °C and 1-min elongation at 72 °C. After 35 cycles, extension was performed for 5 min at 72 °C. Amplified products were visualized under UV illumination with Syber Safe ® staining after electrophoresis using a 1.5 % agarose gel. PCR products were cloned by the pGEM® -T Easy Vector System Kit according to the manufacturer’s instructions (Promega, Lyon, France). They were sequenced in both directions using the Big Dye® Terminator V1.1 Cycle Sequencing Kit (Applied Biosystems). Original sequences have been submitted to GenBank.

### Sequence alignment and phylogenetic analysis

Sequencing products were resolved using an ABI PRISM 3130 automated sequencer (Applied Biosystems). Sequences were compared with the GenBank database using the online BLAST program (www.ncbi.nlm.nih.gov). The highest percentage of sequence similarity was used to identify isolates. Sequence similarity higher than 97 % with a described species was considered to be indicative of identification at the species level. Phylogenetic analysis was established by the neighbor-joining method using MEGA5 software (www.megasoftware.net). Phylogenetic construct was based on the 18S rRNA gene sequences aligned with 52 references.

## Results

### Free living protozoa

A total of 20/233 (8.6 %) CL solution specimens collected between 2009 and 2014 from 16 patients, cultured at least one free-living protozoa (Table 1). Protozoa identifications were made by partial sequencing of the 18S rRNA gene and by establishing the percentage of similarity of these sequences with reference sequences. authenticated by the validity of positive and negative controls. With one exception, confident identification was obtained at the genus level only. These identifications include *Acanthamoeba* in 16 (80 %) solution specimens collected from 12 different patients, *Colpoda steini* in specimen n°14, *Cercozoa* sp. in specimen n°12, *Protostelium* sp. in specimen n°15, and an identical 99 % sequence similarity with both *Hartmanella* and *Vermamoeba* genus in specimen 13.

**Table 1:** List of protozoa identified in 16 contact lens solution specimens, along with co-cultured bacteria and fungi

Patient	CL case	Protozoa	Co-cultured bacteria	Co-cultured fungi
Patient 1	1	*Acanthamoeba* sp.	*Serratia liquefaciens* *Stenotrophomonas maltophilia* *Pseudomonas aeruginosa*	None
Patient 2	2	*Acanthamoeba* sp.	*Pseudomonas aeruginosa* *Stenotrophomonas maltophila* *Chryseobacterium dacguense* *Citrobacter freundi*	*Sacrocadium kiliense*
Patient 3	3	*Acanthamoeba* sp.	*Pseudomonas aeruginosa* *Chryseobacterium gleum* *Delftia acidovorans*	None
Patient 4	4	*Acanthamoeba* sp.	*Pseudomonas fluorescens* *Mycobacterium chimaera* *Stenotrophomonas maltophila*	None
Patient 5	5	*Acanthamoeba* sp.	None	None
Patient 6	6-1	*Acanthamoeba* sp.	None	None
	6-2	*Acanthamoeba* sp.	None	None
Patient 7	7	*Acanthamoeba* sp.	None	*Candida guilliermondii* *Fusarium oxyporum*
Patient 8	8	*Acanthamoeba* sp.	*Stenotrophomonas maltophilia* *Raoultella ornithinolytica* *Sphingobacterium multivorium* *Agrobacterium tumefaciens* *Klebsiella terrigena* *Pseudomonas hibiscicola* *Shewanella putrefaciens* *Sphingobacterium siyangense*	None
Patient 9	9-1	*Acanthamoeba* sp.	None	None
	9-2	*Acanthamoeba* sp.	None	None
Patient 10	10	*Acanthamoeba* sp.	*Klebsiella pneumonia* *Enterobacter cloacae* *Stenotrophomonas maltophila*	*Candida parapsilosis* *Candida lipolytica*
Patient 11	11-1	*Acanthamoeba* sp.	*Sphingobacterium multivorum* *Aeromonas veronii* *Aeromonas caviae* *Raoutella ornitolytica* *Klebsiella pneumoniae*	None
	11-2	*Acanthamoeba* sp.	*Pseudochrobactrum asaccharolyticum* *Aeromonas caviae* *Wausteriella falsenii*	None
Patient 12	12	*Cercozoa* sp.	*Klebsiella oxytoca* *Stenotrophomonas maltophila* *Alcaligenes xylosidans* *Pseudomonas aeruginosa*	*Candida colliculosa*
Patient 13	13	*Vermamoeba* sp.	*Enterobacter cloacae,* *Stenotrophomonas maltophila* *Xanthobacter flavus* *Pseudomona aerouginosa* *Mycobacterium chelonae*	None
Patient 14	14	*Colpoda steini*	None	None
Patient 15	15	*Protostelium* sp.	*Alcaligenes xylosoxidans* *Stenotrophomonas maltophila* *Pseudomonas aeruginosa* *Sphingomonas multivorum* *Aeromonas culicicola* *Hicrobacterium flavum* *Chryseobacterium hominis* *Microbacterium testaceum*	None
Patient 16	16-1	*Acanthamoeba* sp.	*Microbacterium oxydans*	*Penicillium chrysogenum, Candida parapsilosis* *Fusarium oxysporum*
	16-2	*Acanthamoeba* sp.	None	None

**Fig. 1:**
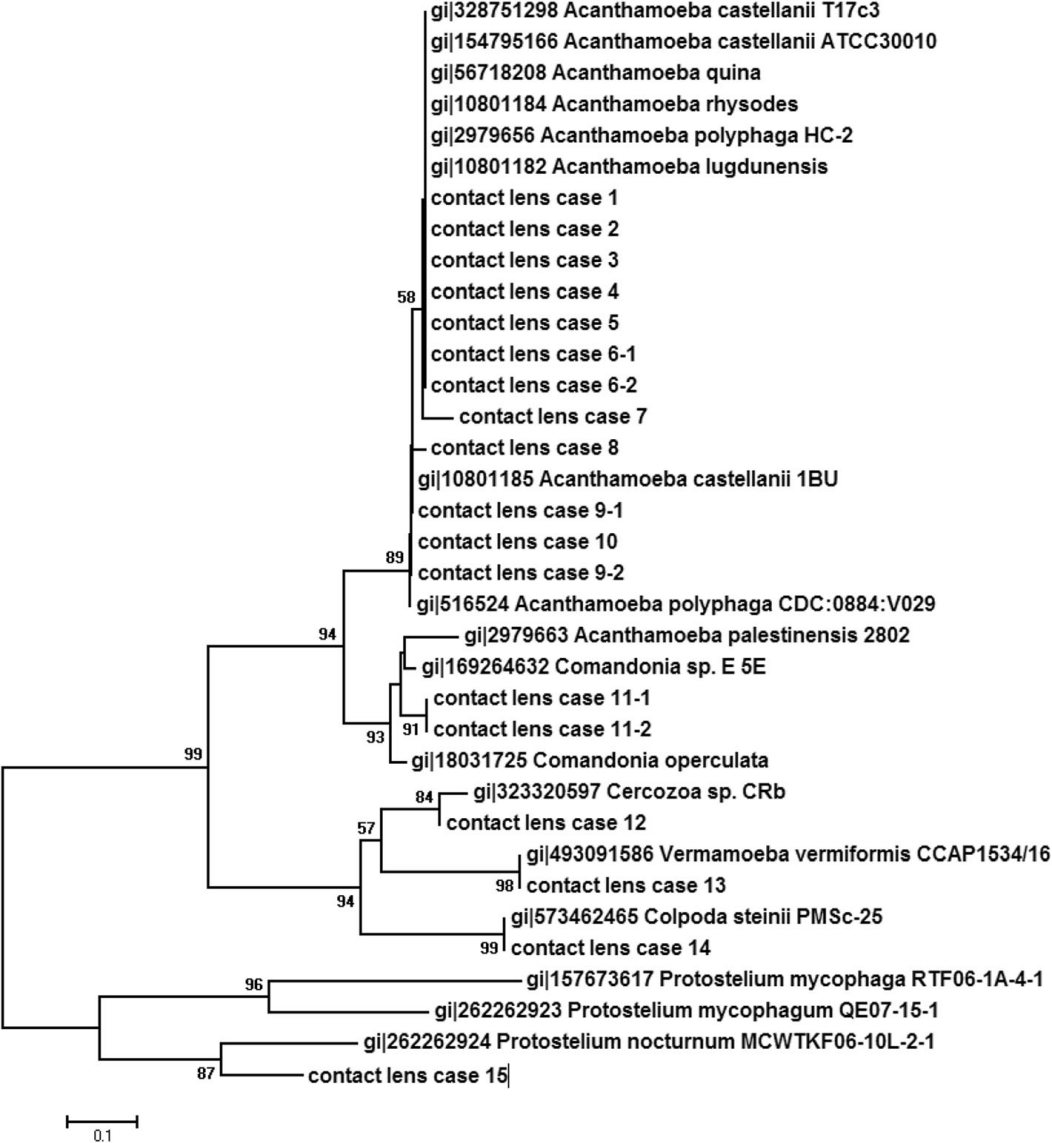
Phylogenetic tree based on the 18S rRNA gene sequences derived from 20 protozoan isolates taken from contact lens solutions. Bootstrap values are indicted at nodes. The *bar* indicates 1‰ substitutions in sequences

Further phylogenetic analysis (Fig. 1) confirmed these identifications and indicated that the protozoa isolated in specimen n°13 was more closely related to *Vermamoeba*. Furthermore, phylogenetic analysis indicated that the same *Acanthamoeba* was isolated in left and right contact lens solutions in patients 6, 11 and 16.

### Bacteria and fungi

Twelve of the 20 protozoa-positive (60 %) CL specimens cultured bacteria, while eight protozoa-positive CL specimens did not. *Stenotrophomonas* sp. and *Pseudomonas* sp. were most frequently identified and found in 8/20 (40 %) specimens, followed by *Klebsiella* sp. in 4/20 (20 %) specimens, *Aeromonas* sp. in 3/20 (15 %) specimens, *Chryseobacterium* sp. and *Sphingobacterium* sp*.* in 2/20 (10 %) specimens and *Achromobacter* sp., *Agrobacterium* sp., *Alcaligenes* sp*.*, *Citrobacter* sp.*, Delftia* sp., *Enterobacter sp., Microbacterium* sp., *Mycobacterium* sp., *Raoultella* sp., *Serratia* sp., *Shewanella* sp*.* and *Wautersiella* sp. in 1/20 specimens. Fungi were cultured in five protozoa-positive CL specimens. Fungi included *Candida guilliermondii*, *Candida parapsilosis*, *Candida lipolytica* and *Candida colliculosa*, *Fusarium oxyoprum*, *Sacrocadium kiliense* and *Penicillium chrysogenum.* In three cases, several fungi were co-cultured, including *P. chrysogenum, C. parapsilosis* and *F. oxysporum* in case 16–1, *C. guilliermondii* and *F. oxyoprum* in case 7 and *C. parapsilosis* and *C. lipolytica* in case 10.

## Discussion

We embarked upon a prospective study of the repertoire of free-living protozoa in the CL solutions. In this study we observed that, unsurprisingly, the vast majority of positive specimens grew an *Acanthamoeba* amoeba. A previous study reported 28 *Acanthamoeba* isolates from CL solutions, including *A. lugdunensis, A. hatchettii* and *A. castellani* [4]. Further species were later found to contaminate CL solutions of residents in Southern Korea [5]. Also, amoeba morphologically identified as *A. rhysodes, A. polyphaga* and *A. hatchetti* were reported in CL specimens of patients with clinical keratitis in Austria [8]. Here, we additionally observed that it is most likely that the same amoeba contaminates both the right and the left CL solutions. These observations are of clinical interest, as *Acanthamoeba* are known to cause keratitis [9–12].

However, we failed to find *Colpoda* sp*., Protostelium* sp*.,* and *Vermamoeba* sp*.* in these CL solutions. Likewise, we found no cases of keratitis which were due to any of these three species: non-*Acanthamoeba* keratitis were found to be due to *Valkampfia* and *Hartmanella* amoeba [13, 14].

Amoeba, and *Acanthamoeba* in particular, have been shown to host so-called amoeba-resisting bacteria [15, 16], making them a source of polymicrobial keratitis which may involve the amoeba itself in addition to bacteria and viruses [17]. Several bacteria here co-cultivated with *Acanthamoeba*, are amoeba-resisting bacteria, including *P. aeruginosa* [18] *Mycobacterium* sp. [19–21] and *Aeromonas* sp. [16, 22]. We also co-cultivated several bacteria with *Cercozoa* sp., *Vermamoeba* sp. and *Protostelium* sp., but not with *C. steini*, suggesting further studies of the relationships between these protozoa and bacteria may be required.

## Conclusions

In conclusion, the spectrum of protozoa contaminating CL solutions is broader than previously thought. These protozoa may also host ocular pathogens including bacteria and fungi. Some of these emerging protozoa escape the current routine detection of amoeba in clinical specimens collected from corneal lesions, underscoring the need to develop additional laboratory tools for the diagnosis of keratitis.
